# Digital bioethics: introducing new methods for the study of bioethical issues

**DOI:** 10.1136/medethics-2021-107387

**Published:** 2021-09-11

**Authors:** Manuel Schneider, Effy Vayena, Alessandro Blasimme

**Affiliations:** Health Ethics and Policy Lab, Department of Health Sciences and Technology, ETH Zurich, Zurich, Switzerland

**Keywords:** ethics, information technology, scientific research

## Abstract

The online space has become a digital public square, where individuals interact and share ideas on the most trivial to the most serious of matters, including discussions of controversial ethical issues in science, technology and medicine. In the last decade, new disciplines like computational social science and social data science have created methods to collect and analyse such data that have considerably expanded the scope of social science research. Empirical bioethics can benefit from the integration of such digital methods to investigate novel digital phenomena and trace how bioethical issues take shape online.

Here, using concrete examples, we demonstrate how novel methods based on digital approaches in the social sciences can be used effectively in the domain of bioethics. We show that a digital turn in bioethics research aligns with the established aims of empirical bioethics, integrating with normative analysis and expanding the scope of the discipline, thus offering ways to reinforce the capacity of bioethics to tackle the increasing complexity of present-day ethical issues in science and technology. We propose to call this domain of research in bioethics *digital bioethics*.

## I. Bioethics research in a digital world

Since the late 1960s, bioethics has steadily established itself as a novel form of expertise offering theoretical insight and normative guidance on philosophical, ethical, social and legal issues emerging from advances in medicine and the life sciences.^1^

In addition to philosophical analysis of prominent bioethical issues, social science methods have increasingly been employed in the field of bioethics over the last two decades, giving rise to a new area of inquiry known as empirical bioethics.^2^ While the relation between normative and empirical bioethics remains a matter of debate, the importance of empirical inquiry for bioethics is widely recognised.^3^ In this paper, we argue that digital methods can expand the capabilities of empirical bioethics by turning the online space into the object of robust empirical research.

With the rise of the World Wide Web at the end of the last century, the digital infosphere emerged as a space where humans exchange ideas and knowledge, giving rise to novel forms of discourse. These online spaces were not mere digital realisations of analogue counterparts but truly novel phenomena that could only materialise and exist in the digital form.^4^ Around the turn of the millennium, social scientists acknowledged the unique nature of digital spaces and began to investigate them as such.^5^ This novel area of research prompted the development of new research methods. Some of these methods were digitised versions of existing tools, such as web-based surveys.^6^ Others aimed to capture the specific ways in which knowledge and opinions take shape online. These include computer-assisted methods to reveal and analyse the networked structure of online content and the interaction between digital entities such as websites (for instance, via the automated analysis of hyperlink structures).^7^

In the present day, as human interactions increasingly take place online, most individuals enthusiastically espouse what Langdon Winner called ‘electronically mediated forms of living’.^8^ More and more, people spend time online every day and connect to one another on social media platforms. As a consequence, the online space has become a digital agora where individuals interact and share ideas on all sorts of matters. As Boellstroff and colleagues put it in the introduction to their handbook on digital ethnography^9^:

‘Virtual worlds are places of imagination that encompass practices of play, performance, creativity, and ritual. The social lifeworlds that emerge within them are very real. […]. They draw upon physical world cultures in multiple ways yet at the same time create possibilities for the emergence of new cultures and practices. Just as in the physical world, people within virtual worlds perform and cycle through different roles and identities’.

While many social science domains have been using the online world as a site of rigorous research for some time, empirical bioethics has not yet pursued dedicated methods and standards to address bioethical issues online.

Throughout this paper, we understand the word *digital* in an ontological sense, as ‘all that has been developed by, or can be reduced to, binary code’.^10^ While we call attention to the importance of digital worlds as sites of empirical research in bioethics, our work does not imply that digitally mediated forms of discourse and agency are more relevant than their analogue or predigital counterparts. In particular, we do not take digital technologies to produce meaning and forms of agency independent of analogue forms of culture. Rather, drawing on what appears to be a consensual view in Internet studies and digital anthropology, we take digital cultures to be in a dialectic and cross-dependent relation with analogue ones.^11^

In section II, we offer an overview of empirical bioethics, including its disciplinary status and methods. Section III discusses novel methodological approaches to the study of the online world that are emerging in fields such as computational social sciences. In section IV, we propose a definition of digital bioethics methods and illustrate the potential of these methods as developed and integrated in our research activities. Finally, in section V, we discuss challenges and opportunities associated with the uptake of digital bioethics and suggest ways to overcome potential barriers to its future growth.

## II. Empirical bioethics

When bioethics first emerged as a discipline in the late 1960s, most scholars in the field had a philosophical, theological or legal background.^12^ Bioethical scholarship thus revolved mostly around definitional issues (such as the definition of disease, death or competence). Its methods consisted mainly of philosophical analysis, and its aims focused on providing reasonable ways to support decision making in medicine and science.^12^ While the role of philosophy remains highly relevant today, insight from other disciplines such as sociology, history, policy, science and medicine have gained significant recognition.^1 12 13^

Two decades ago, as an increasing number of social scientists began to engage with bioethical issues, an empirical turn in bioethics research has occurred.^2 14^ The interdisciplinary characteristics of the field easily accommodated these novel contributions.^15 16^ However, the theoretical relation between normative and empirical bioethics was, and remains, a matter of debate.^2 17^

Empirical bioethics employs social science methods to collect quantitative and qualitative data about bioethical issues and related stakeholders’ views and attitudes. One primary contribution of empirical bioethics has thus consisted in giving a voice to ethical concerns around controversial issues in medicine, science and technology.^3^ In this respect, empirical bioethics has provided insight into the ways ethical issues relate to their specific sociological, political and cultural contexts, as well as how various actors articulate their perspectives around a given ethical question.^2^

The question of whether and how insight from empirical bioethics can directly contribute to normative analysis and decision making remains unsettled. The distinction between matters of fact and matters of value has a long philosophical genealogy, conventionally dating back to David Hume’s thesis that one cannot derive ‘ought’ from ‘is’,^18^ ^i^ to more recent metaethical debates about the merits of ethical naturalism.^19^ A dichotomous understanding of the relation between facts and norms (‘is’ and ‘ought’) has to some extent prevented the seamless integration of empirical methods into normative bioethics and decision making.^ii^ This condition has resulted in academic tension between the two fields,^20 21^ with some scholars viewing empirical work as mainly descriptive, while others consider it conducive to reorienting action-guiding norms in a practical (normative) sense.

The tension between empiricism and rationalism in bioethics, however, has also been a productive one, in particular as it enabled scholars to flesh out the limitations of philosophical bioethics and to reconcile moral theories and normative analyses with empirical evidence as well as with the lived experience of moral agents.^24^

The objectives of empirical bioethics studies span a continuum between descriptive, evaluative and normative.^25^ As Kon^26^ noted in 2009, some empirical bioethics studies are indeed descriptive, seeking ‘to define current practices, opinions, beliefs, or other aspects that may be considered the status quo’. This strand in bioethics—that Kon calls ‘lay of the land’ research—offers empirical insight into the preferences, expectations and values of patients, caregivers, nurses and physicians but also biomedical researchers and research participants. The same category includes studies aimed at offering empirical knowledge in support of normative considerations or decision making, as in the case of knowledge about the quality of life of patients on life support systems. Another strand of empirical bioethics seeks to shed light on the extent to which a given ethical norm is actually followed in clinical or research practice. Kon calls that ‘ideal vs reality’ research,^26^ and it includes studies aimed at probing compliance with both procedural norms (such as informed consent) and moral values (such as justice and equity in access to healthcare). Some empirical bioethics studies also employ experimental approaches to test whether ethical norms and principles are effectively implmented^27^ and to measure their impact on patient care and research practices.^26^ Such lines of research—that Kon classifies under the rubric ‘improving care’—attempt to realign ethical norms with clinical or scientific reality in a specific context.^3 26^

Closer to the normative end of the spectrum, a further aim of empirical bioethics is to critically discuss or even changing existing ethical norms probing the robustness of their empirical premises or assumptions, for instance, regarding what pertinent stakeholders value the most from an ethical point of view. The aim here—as Kon also observes—is not to make moral analysis dependent on subjective opinion, but rather to ensure that what we consider morally appropriate actions is informed by empirical work about what people actually value and have reason to value.^26^

Finally, empirical research in bioethics is also devoted to the study of the institutions of bioethics (such as clinical ethics committees, ethics review committees and national bioethics commissions) from a sociological point of view.^25 26^

To accomplish such a varied set of disciplinary aims, empirical bioethics researchers have borrowed methods from other social science disciplines, such as sociology or anthropology. Thus,the toolbox of empirical bioethics includes cross-sectional surveys, scenarios, empirical reviews, ethnographies, semistructured interviews, focus groups, qualitative content analysis, public opinion polls and deliberative methods.^23 28^ Both quantitative and qualitative data can be used in an empirical bioethics study, and quite often the two approaches are combined.

### Expanding the empirical gaze

The debate about the aim of different methodological approaches in empirical bioethics has been intense over the course of the last couple of decades.^17^ Different methods enable different forms of integration between empirical insight and normative conclusions, depending on whether normative conclusions are to be justified as a matter of consensus or logical argument, whether they depend on processes such as inclusive deliberation or whether such conclusions should be generalisable or limited to a given context of practice.^29^

Empirical bioethics, however, has only begun to devote specific attention to methods that could be employed to make sense of the way bioethically relevant issues are discursively articulated online.^30^ The online space is not simply an electronic repository of information. As we mentioned in the introduction, the digital sphere is best understood as a platform of communication and agency existing in a mutually productive relation with analogue cultures and forms of interaction. Over time, this view of the digital as a material culture became prominent in the social sciences. As Horst and Miller^10^ wrote, digital worlds ‘are neither more nor less material than the worlds that preceded them. […] This concerns humanity’s remarkable capacity to reimpose normativity just as quickly as digital technologies create conditions for change’.

With the rise of the Internet in the mid-1990s, digital networks enabled new forms of communication and interaction. In the span of two decades, the internet evolved from a communication infrastructure to a ‘participatory web’, in which users take an active role creating content and shaping the world wide web, rather than using it as simply an information repository or communication platform.^31 32^

Missing out on the online space as a site of rigorous empirical research precludes access to influential spheres of discourse and practice that have direct bearing on bioethical analysis and decision making. Digital methods, on the other hand, enable empirical bioethics to document how ethical values and perspectives are articulated online, which principles, interests and intuitions are mobilised and how their relevance fluctuates over time.

However, the value of embracing digital methods in bioethics lies beyond their use in ‘lay of the land’ research. Expanding the reach of bioethics to the online space can foster integrative analysis that fruitfully combine normative and empirical research. More specifically, digital methods can contribute to both normative analysis and the implementation of bioethically relevant norms in practical contexts. As to the former, by looking at how ethically relevant matters are discussed online, digital methods enable probing bioethical arguments based on assumptions about the interests, values and views of different publics. This capacity can also be used to detect areas of overlapping consensus between different stakeholders so as to advance normative conclusions of a pragmatic character.^29 33^

Moreover, digital methods can detect the emergence of novel normative discourses before they become embedded in mainstream ethical narratives about ongoing controversies. The web is a space of connectedness where bonds are formed and nurtured among people with shared interests and stakes. For this reason, digital methods can be particularly useful to shed light on otherwise disfranchised discursive communities and on the way they spontaneously articulate their moral experience in relation to bioethically relevant issues.^3^

As we show below, digital methods can reconstruct how moral attitudes of the public or specific stakeholders evolve over time—for instance on social media platforms—enabling the analysis of what triggers people to acquire and manifest publicly their moral attitudes in an issue of bioethical interest. This is possible thanks to the fact that users of social media platforms leave traces of their past interactions that are accessible through computational methods.

Considering integration between empirical and normative bioethics at a more practical level, digital methods can be employed to provide what Mildred Solomon^3^ has called a ‘bridge between conceiving a moral vision of a better world, and actually enacting it’. In particular, digital methods can reveal how different institutional actors relate to one another as to their online presence enabling interpretive insight into potential impediments due, for instance, to insufficient harmonisation of normative requirements.

We thus suggest that digital methods can be fruitfully employed in empirical bioethics as they shed new light the reality of discursive practices as they take shape in the online space.

In the next section, we explore methods developed by other social science disciplines for the analysis of digital discourse and human interaction online, before turning to illustrate digital methods we have developed and employed specifically in the domain of bioethics.

## III. Digital methods for the social sciences

At the beginning of the 2000s, social scientists working in domains such as anthropology and museum studies had begun to identify digitally mediated forms of communication and agency as fascinating areas for empirical research.^4^

Specific social science methods were developed to enable analysis of novel phenomena taking place online and through digital platforms.^5^ Ethnographers in particular were among the first to devote attention to the online world and how it is imagined by its designers and lived by its users, both as individuals and as groups or communities.^9 10^ Digital ethnography explored ways in which the virtual enables users to assume roles and identities, to link with others, and to spread opinions, judgments of value, bits of knowledge and worldviews. Snee and colleagues^34^ define such ‘digital methods’ as ‘the use of online and digital technologies to collect and analyse research data’.

A variety of digital methods have been developed over the years.^35^ On one hand, well-established empirical methods have acquired a digital format, epitomised by web surveys and online focus groups.^6 36^ On the other hand, computer scientists, physicists and social scientists have come together to develop automated approaches for the analysis of massive amounts of data, giving rise to what is now called computational social science.^37^ Such methods include computer-mediated discourse analysis,^38^ the investigation of online hyperlink structures^7^ and the analysis of Global Positioning System (GPS) data.^37^ Natural language processing (NLP) enables the study of textual data sets, such as website contents and social media posts that, due to their volume, would not be accessible via manual content analysis. Recently, machine learning approaches have entered the spotlight, and huge language models with billions of parameters promise a revolution in the understanding of human language.^39^ Given its reliance on big data sets, the establishment of robust data mining methods has been instrumental in the growth of this field.^40^

A close relative of computational social science, social data science is interested in the analysis and visualisation of digital social traces through data.^41^ With the emergence of social networks such as Facebook, Twitter and Gab, the study of social media platforms became an interesting research subject and a promising data source, due to the abundance of digital traces left by users on a daily basis.^42^ Through social media analysis, researchers can study the interaction and relationship between users and their opinions expressed online, attitudes towards a certain topic and more.^43^

Methods from social network analysis have been successfully applied to a variety of research areas and allow for the study of social phenomena through connections between actors, such as friendship relations or geographic proximity.^44 45^ Social network analysis uses descriptive statistics as well as modelling techniques that allow insights into actors, groups of actors, the influence of relationships between actors and groups and which variables might explain the observed properties and dynamics. Thanks to the development of tools such as Gephi that enable the computation of relevant statistics and visual exploration of \ networks through a graphical user interface,^46^ network analysis has become more accessible even to researchers without specific technical knowhow.

## Digital bioethics methods

As we observed above, an ever-increasing portion of bioethically relevant discourse takes place online. Social media platform users comment on complex issues in science and technology, such as ethical limits and governance tools needed for new genetic engineering technologies such as CRISPR-Cas9 (see further), or public health ethics issues such as the use of vaccines. Reconstructing the thematic articulation and the networked structure of online debates can shed light on the way ethically loaded narratives emerge and diffuse. Moreover, studying the relations between digital objects, such as ethical guidelines issued by international organisations and scientific society, can illuminate areas of consensus and divergence and justify calls for policy initiatives. Analysing the digital links between different organisations with the aid of social network analysis can lead us to probe the impact and reputation of stakeholders involved in a given emerging field of science and technology and therefore to identify key domains for normative analysis.

New communities continuously form and interact online, offering expanded opportunities for self-identifying practices and patient empowerment. On platforms like Open Humans, for example, people interested in open sharing of their genomic data can join a community that both shapes and puts into practice individual views about personal genetic data.^47^ The project Patient Innovation is another such example, supporting patients to connect online and enabling patient driven innovation.^48^

Therefore, the use of digital methods for bioethics should not be restricted to the study of online cultures, that is, consideration of the online world and its relation to the offline world as an object of study. Rather it should expand to include the use of computational techniques that treat online content and the digital traces of online activities as sources of knowledge^49^ and as the basis of knowledge claims important to the advancement of normative insight and policy analysis.

We present four native digital methods for empirical bioethics research that we have developed and implemented in published studies (see table 1 for an overview)^50 51^: a sentiment analysis of tweets on CRISPR/Cas genome editing using machine learning (online sentiment analysis); an analysis of topic trends through hashtag statistics (automated topic ranking); the identification of themes from existing studies on attitudes and concerns towards gene editing in tweets (thematic probing); and a network analysis of international data sharing policies (policy landscape analysis).

**Table 1:** Overview of the four methods we developed and employed in our studies

Method	Online sentiment analysis	Automated topic ranking	Thematic probing	Policy landscape analysis
Aim	Understanding public attitudes towards a subject over time	Identifying main topics of online discussions	Probe the occurrence of predefined themes in online discussions	Building hypothesis on online policy documents
Related methods	Public opinion polls	Qualitative content analysis	Qualitative content analysis	Social network analysis
Technical approach	Natural language processing	Statistical big data analysis	Regular expression matching	Crawling, network analysis

### Online sentiment analysis

In a study conducted in 2018 and 2019, we examined public attitudes towards CRISPR/Cas-9 gene editing through an analysis of social media content posted by Twitter users.^51^ We acquired a little over 1.5 million tweets containing the keyword CRISPR through GNIP, a Twitter subsidiary and the official Twitter Application Programming Interface (API) for a 6.5-year period. We used a machine learning approach based on NLP to determine the sentiment (positive, neutral and negative) in each of the tweets: a sample of the tweets was labelled through the platform ‘crowdbreaks’ and multiple machine learning models were trained using that sample.^52^ The type of machine learning model that performed best on a test set was a fairly new type of NLP model called BERT,^53^ first published in 2018. The trained model was then used to predict the sentiment of the remaining tweets, and a daily sentiment signal with a rolling average of 7 days was calculated, resulting in a sentiment index between −1 (all negative) and 1 (all positive). Using a second classifier trained on the tweet sample, we classified all tweets according to the organism the tweet referred to (e.g. humans, animals, plants etc.) and made a breakdown of the sentiment per organism class over time. The results revealed a generally positive sentiment with a series of negative spikes in the second half of the analysed period and a shift in the general sentiment dependent on the organism class.^51^ The detection of such discrete changes in ethical sentiment points to a causally relevant role that can be attributed to specific uses of a given technology or to specific advances in basic or applied science. In the case of CRISPR, for instance, the spikes correlate with the announcement, in late 2018, of clinical uses of gene editing to modify the genome of two human embryos eventually resulting in the birth of two baby girls.^54^ This kind of analysis can help identify precisely which ethical lines the public is uncomfortable with being crossed, which in turn can lead to better calibration of normative arguments and socially more robust decision making around controversial forms of scientific innovation.

### Automated topic ranking

In the same study, we analysed the hashtags used in tweets to uncover which topics were most discussed on Twitter. For this method, we used statistics rather than machine learning to see how frequently a hashtag was used per year, which allowed us to see how often the tweets referred to certain topics in each year, that is, the number of tweets containing the corresponding hashtags. The hashtags are particularly useful since they represent labels directly provided by users themselves, for their own statements. Topics were ranked according to their counts, revealing the most discussed topics for each year and how the prominence of a topic changed over time. This topic ranking allowed us to see, for example, the point at which the conversation on the birth of two genetically modified babies in China in late 2018 picked up, and how the prevalence of other topics changed at the same time. Furthermore, linking this topic breakdown to the sentiment of the corresponding tweets allowed us to observe how the sentiment of Twitter users towards that specific topic changed over time.

Sentiment analysis and automated topic ranking employ advanced technical methodologies to large-scale analysis of moral attitudes on divisive public policy issues in bioethics. Moreover, such methods have a remarkable explanatory potential as they enable to observe how ethical views evolve over time and in connection to specific scientific advances or controversial uses of discoveries and technologies. More specifically, analysing hashtags provides a dynamic picture of the relative relevance that the public attributes to different moral concerns. Such a dynamic picture can reveal normative trade-offs between different values and principles, and it can as well drive moral interpretation for instance suggesting ways to balance and specify different ethical principles in specific context.^55–57^

### Thematic probing

Understanding public opinion on gene editing in humans was the focus of multiple studies prior to our study on CRISPR. This offered the opportunity to use digital methods to connect our study with results obtained through conventional surveys and to assess our sentiment analysis in a context beyond Twitter. To that end, we first identified existing studies about public opinion on human gene editing and used an inductive coding approach to identify themes occurring in these studies. The themes were then translated into regular expressions, that is, patterns that describe which character sequences in a text are matched or not,^58^ which enabled probing of the textual data from the tweets for occurrence of these themes. Thematic probing allowed us to identify which of the themes from the survey results were also present in the conversation on Twitter (ie, the corresponding regular expression was matched in the text of the tweets), what portion of the conversation referred to a specific theme and which sentiments were associated with these themes over the years. This process is similar to a qualitative content analysis with a predefined codebook but allows for probing of a much larger dataset. Moreover, thematic probing enables comparative analysis of online and offline discourses ushering in opportunities for novel insight into how ethical views emerge and diffuses on different communication platforms. Understanding both discrepancies and areas of overlap between online and offline discourses can shed light on different factors that influence moral views. This is useful because it can illuminate the persuasive potential of moral arguments in different contexts. Moreover, as for the previous methods, thematic probing can suggest ways to interpret prima facie norms and balance principles based on their relative weight.

### Policy landscape analysis

Another digital method that we developed entailed building research hypotheses to be further investigated with the ultimate aim of generating normative insights for policy and decision making. In a study conducted in 2017,^50^ we used network analysis to build a hypothesis on existing data sharing policies published online. First, 230 policy documents were identified through a manual online search procedure. We used a digital method to detect the hyperlink structure among the online locations (uniform resource locators or URLs) of sites containing policies or hosting policy documents. We used both a database (moz.org) providing backlinks to policy sites, as well as a web crawler to identify chains of hyperlinks pointing to the policy sites. This procedure resulted in a hyperlink network consisting of connections among the identified policy documents. Using network analysis, we measured the length of the shortest path between policies (ie, the minimum number of steps required to navigate from one policy document to another) and the density of strong network components and found that the our documents set formed only a sparsely connected network. This indicated that organisations working to promote data sharing for research purposes might have developed their guidance in relative isolation from one another. This result suggested that the promotion of data sharing, while widely supported, might suffer from a lack of harmonisation over ethically relevant principles and, on a practical level, inadequate standards to improve data sharing in practice. To test this hypothesis, we performed a conventional qualitative content analysis (with the aid of the NVivo software), resulting in a set of recommendations for how to address bottlenecks hindering efficient data sharing.^50^

Network analysis enabled a novel understanding of the relationship between different stakeholders (in this case, policy actors and expert bodies) and provided us with insight into the possible explanation for inefficient implementation of ethical norms relative to data sharing practices in the research context. In particular, it enabled us to observe that the actors promoting data sharing guidance form only a loosely connected network online, suggesting a fragmented policy landscape as a possible explanation of why such policies fell short of producing their expected outcome. We further probed this hypothesis by detecting a rather diverse and unfocused set of ethical recommendations in this area. This method focuses primarily on institutional actors and how they relate discursively to one another,^25^ and it proved useful to drive normative analysis and ethical recommendations.

## V. Implementing digital bioethics

The digital methods illustrated above are both qualitative and quantitative and have been used in conjunction with other methods (mostly digitised version of conventional empirical methods) routinely applied in empirical bioethics. Nevertheless, we illustrated four *natively* digital methods instead of digitised versions of empirical methods. Such digital methods depend essentially on computational techniques, rather than methods that simply migrated to digital format for practical reasons.^49^ Nevertheless, this analytic distinction does not imply that native and digitised methods cannot be used in conjunction in the context of the same study or that they cannot be integrated with more conventional analogic methods based on study-specific requirements.

In this respect, our aim is not to attempt to replace analogic methods with digital ones. Rather we have presented tools to further expand the scope and the reach of empirical research in bioethics. Our approach assumes the possibility of fruitful integration of empirical insight into normative analysis and development of practical action-guiding norms. In other words, in line with most scholarship in empirical bioethics, we do not endorse a sharp separation between descriptive and normative bioethics. To the contrary, we see digital methods alongside non-digital ones as valuable aids for normative bioethics, and we support the prospects of a productive integration between the two fields. While we are aware of the theoretical as well as practical challenges of an integrative approach,^59^ our initial steps in the creation of digital bioethics methods demonstrate that the prospect of integration is a realistic and productive one.

Digital methods are to be understood as tools to observe a range of discursive practices happening online. However, the ultimate aim of such research orientation is not just to offer a descriptive account of those practices, but to connect them to the offline reality of controversial issues in bioethics. As we said above, rather than looking at online and offline discourse as two separate worlds, or taking the former to be a simple mirror of the latter, we assume a mutually productive relationship between the two.

### Added value

Empirical methods of the kind we have proposed here allow research to gain access to novel sites in which different stakeholders (both individual and institutional) express their moral ideals, declare their interests and share their views as to the norms and principles that should guide action in bioethically controversial matters. This is consistent with a common integrative understanding of empirical bioethics and cognate disciplines (see, for instance, refs 25 and 29). However, at the same time, digital methods hold promise to expand the explanatory power of empirical bioethics. In particular, methods to trace the evolution of moral views over time (like sentiment analysis and automated topic ranking) enable a fine-grained evaluation of what triggers certain moral reactions in the public. This can have direct implications for normative analysis, for example, by challenging value judgements about what is—in a given bioethical controversy—that should be deemed worth of ethical consideration.

The relationship between online and offline worlds is complex and mutually productive, but a lot of work is still needed to clearly understand how they influence each other, under which circumstances and to which extent. Methods like thematic probing offer explanatory insight into such dynamics. Finally, a method like the analysis of policy landscapes offers the opportunity to explain how different institutional actors, such as policy-making bodies determine the implementation (or lack thereof) of ethical norms in a given domain of practice, with implications on how best to address potential bottlenecks from a normative point of view.

The methods we have introduced seem particularly useful in the context of bioethical issues reaching the status of public controversies or topics that lend themselves to specific forms of decision making by institutional bodies and actors. Nevertheless, it can be expected that, in the future, computational methods will be employed also in the context of medical ethics, where ethical decision making is of a more particular and context-specific character.

### Technical challenges

The successful integration of our new digital bioethics methods into empirical and—in turn—normative bioethics depends on a variety of technical factors and factors concerning the disciplinary constitution of the different fields.

Technically, we ourselves encountered several obstacles that could hinder other researchers employing similar methods. In our analysis of the data sharing policy landscape, for example, different URLs can lead to the same object. While, for example, different character encodings (ie, the technical representation in 0s and 1s, which can be done in different ways) can be detected rather easily, assessment of the equality of two targets might only be possible by comparing the contents of the two webpages. A further challenge resulted from the sheer number of webpages and the even greater number of hyperlinks between them. Finding the shortest path from one webpage to another using manual methods is virtually impossible, and even in employing a web crawler, the time needed to explore several steps of hyperlinks is significant and grows exponentially with the number of steps and corresponding number of websites. We used a backlink service (moz.org) that provided the URLs of webpages that point to the policy webpages, in order to reduce the number of websites from which we needed to extract hyperlinks. However, we could only make limited use of this service due to rate limits.

### Cost of data

An additional challenge is presented by the cost of access to data, an issue that we encountered for instance in the Twitter study on CRISPR. While Twitter offers a cost-free API, access to historic data through this API is limited. For our study, we needed to acquire a significant amount of data through the Twitter subsidiary Gnip, which sells historic Twitter data. The cost of these data is significant for a research project, not only in the case of Twitter, but for other data providers as well. Therefore, funding for digital bioethics research must also cover data acquisition costs, for purchasing both archived data and API subscriptions. Alternatively, the scientific community could campaign for Internet data to be freely available for not-for-profit research purposes.

### Standards

The variety of accepted methodologies in empirical bioethics has fostered the distinct interdisciplinary nature of this field of research.^29^ Accordingly, many scholars have called for a more rigorous definition of methodological standards to support the growth of the discipline. Samia Hurst, for instance, points out that bioethics has imported methods from empirical disciplines without absorbing the ‘standards to which researchers in these disciplines are held’.^14^ This concern spans choice of appropriate methodology, data collection practices, data presentation, consistency of empirical conclusions and understanding of study limitations. Ives and colleagues^60^ identified a series of advantages that could be derived from widespread adoption of methodological standards of practice in empirical bioethics research. In particular, such standards would enhance consistency in the assessment of grant proposals and the evaluation of peer-reviewed articles, making such processes more predictable for researchers and more manageable for funders and editors.

With the development of novel digital methods, questions arise over standards and data quality as well.^34^ While social scientists can resort to standards of conventional empirical methods, the question of how these standards can and should be adopted for digital methods is a matter of debate.^49^ This challenge is further pronounced in the case of digital bioethics methods, due to the ongoing discussion surrounding empirical bioethics standards in general.^14 60^ By contrast, many of the technical components used for digital methods (such as machine learning) have established standards and best practices of their own. Therefore, we can and should adhere to these standards when such computational methods are used. However, rapid improvements in fields like machine learning may quickly render present standards obsolete.

### Training and skills

Beyond technical and structural limitations, the use of digital bioethics methods requires a substantial amount of technical skills and expertise. The background of bioethics scholars and researchers does not typically include extensive technical training, and this can limit the choice of digital methods for bioethics research groups. In this respect, the potential of digital methods for empirical bioethics research depends on the willingness of research institutions to further support the kind of interdisciplinarity that has already characterised bioethics over the past two decades. This consideration applies to universities, funding agencies and editorial boards alike. As the importance of digital methods in the social sciences increases, bioethics journals and reviewers must be open to manuscripts employing these methods. Initially, the use of digital methods will not be supported by commonly accepted quality standards, which will arguably complicate the assessment of manuscripts. However, this problem was also present in the early days of empirical bioethics and did not prevent the growth of the field.^14 60^

## Conclusion

By introducing digital methods to empirical bioethics and demonstrating their relevance, we hope to initiate a broader discussion on how bioethics can advance in the digital domain. Although the use of digital methods for empirical bioethics research is still in its infancy, we have shown that their integration into current research practice is fruitful. However, in order for the novel methodological approaches to become entrenched in the toolbox of empirical bioethics, more collaboration and exchange is needed. In particular, community-wide efforts are essential to establish standards for methodologies that, at present, are still to be considered experimental. More dialogue is needed to discuss the merits and limitations of digital methods, overcome technical bottlenecks and standardise research protocols. Nevertheless, as we have illustrated, digital methods thus far have proven valuable for empirical bioethics research and provide an opportunity to begin building *digital bioethics*. We hope this paper will encourage others to build robust methodological standards for digital methods in empirical bioethics research.

## Data Availability

Data are available upon request.

## Footnotes

From a practical point of view, this philosophical position sustains some commonly held ideas about ethics such as that majority opinions do not support normative conclusions, and that what is legal or illegal is not necessarily moral or immoral (see,^23^ chapter 1).

We have to point out that these days, such a rigid distinction between ‘is’ and ‘ought’ has come under attack (ex.^22^). However, reviewing such discussion falls beyond the scope of this paper.
